# Poly-Unsaturated Fatty Acids in ADHD and in Other Neuropsychiatric Conditions: A Multiple Case Presentation

**DOI:** 10.3390/pediatric13020032

**Published:** 2021-05-06

**Authors:** Matteo Chiappedi

**Affiliations:** Developmental Psychopathology Research Unit, IRCCS Mondino Foundation, 27100 Pavia, Italy; matteo.chiappedi@mondino.it

**Keywords:** ADHD, omega3, omega6, PUFAs, neurodevelopmental disorders

## Abstract

Neurodevelopmental disorders are seen quite commonly by general pediatricians. They should be managed with a multi-professional approach. The potential beneficial effect of poly-unsaturated fatty acids (PUFAs) has been reported in recent literature, but guidelines describing their use in everyday practice are still lacking. We describe four cases as examples of the possible integration of a supplementation with PUFAs in the management of four relatively common clinical situations (i.e., children too young to receive pharmacological treatment for ADHD, children with nonspecific neurodevelopmental disorders, children whose parents refuse consent for pharmacological treatment of ADHD, and children for whom methylphenidate is not sufficient to achieve expected results).

## 1. Introduction

According to the DSM-5 [1], neurodevelopmental disorders are “a group of conditions with onset in the developmental period… often before the child enters grade school… characterized by developmental deficits that produce impairments of personal, social, academic, or occupational functioning”. This definition includes a number of well-defined disorders, including e.g., Autism Spectrum Disorder, Intellectual Disability, and Attention-Deficit/Hyperactivity Disorder. It is, however, anticipated the possibility to see in clinical practice a number of patients having signs and symptoms of a neurodevelopmental disorder which cause clinically significant distress or impairment but do not reach the diagnostic threshold for any specific diagnostic class.

The prevalence of these disorder is debated in current literature, but as a whole in Italy (and possibly in all developed countries) their symptoms represent a non-negligible reason for consultation with the general pediatrician and of referral to a child neuropsychiatrist.

All neurodevelopmental disorders require a multi-factorial diagnostic approach and treatment. Key factors are an accurate diagnosis, the involvement of families and the availability of evidence-based interventions [2,3]. Psychopharmacological strategies actually available are considered to be only partially useful to manage some more disturbing symptoms, with the notable exception of ADHD where methylphenidate has proven to be highly effective, at least in the short-to-medium term [4]. As in all other neuropsychiatric disorders, the use of complementary and alternative medicine has been considered [5], more often in association with other therapeutic approaches.

Among others, the use of poly-unsaturated fatty acids (PUFAs) has received a significant attention and has been studied in detail, especially in ADHD [6] but also in other neurodevelopmental disorders [7]. PUFAs are one of the main cellular building blocks. A loss of their balance, due to dietary issues, has been considered as a risk factor for many neuropsychiatric disorders [8]. Actual evidence of a possible role of a supplementation of PUFAs in the treatment of neurodevelopmental disorders has provided promising but not conclusive results [9], possibly due to the use of single fatty acids or of omega-3 fatty acids alone; in fact, given the physiological regulation between the different PUFAs in a dynamic equilibrium, it is possible that the utility of this approach relies on the possibility to obtain or restore a correct balance between the different PUFAs (and, therefore, their optimal functionality) [10]. This equilibrium is one of the factors which is altered as a consequence of neuroinflammation, a factor which as been reported as a major shared mechanisms in neurodevelopmental disorders and in other psychiatric disorders [11]; it is possible that the restoration of the omega-3:omega-6 ration could contribute the reducing the dysfunctional activation of metabolic pathways leading to behavioral symptoms [12]. Moreover, there are no guidelines available regarding the use of PUFAs in everyday pediatric practice.

Considering this framework, four case reports are presented to offer insights about the possible use of PUFAs in different commonly seen clinical conditions.

## 2. Case Reports

### 2.1. Case 1—Too Young to Receive Methylphenidate

G. was first seen by a child neuropsychiatrist when he was four years and seven months old. Their parents stated that their child was always moving, like he was driven by a motor. He was unable to play with peers but also with adults because he could not respect his turn and was frequently asking to change the activity, declaring to be “bored”. He had been described by some parents as “uneducated”, because of his tendency to intrude on others and to talk excessively. This description was also confirmed by his general pediatrician, who added a tendency to have falls and minor injuries due to his lack of attention, combined with a too high level of motor activity and a tendency to underestimate risks taken while making motor activities.

A carefully taken history revealed normal pregnancy and term birth without any significant problem. In the first months of life he was described as “always trying to move”, with reduced sleep necessities and short feeding times (leading to multiple small meals). Developmental milestones were reached at normal age, with a slight tendency to be more skilled in motor performances than in language use. In kindergarten, he was considered far too active, with highly developed gross- but reduced fine-motor skills. No treatment or specific counselling had been offered to him or his family before that moment.

Clinical examination was in line with the description provided by parents and teachers. A cognitive evaluation confirmed a normal functioning, with only a frailty in short-term memory and a significant difficulty in both fixing and maintaining attention. This also offered an explanation to the difficulty in completing requests involving sequential tasks described by the parents during the evaluation.

Kiddie-Schedule for Affective Disorders and Schizophrenia—Present and Lifetime (K-SADS-PL, DSM-5 version) was administered and a diagnosis of Attention-Deficit/Hyperactivity Disorder was confirmed [13]. Conners’ Parent Rating Scales (CPRS) [14] and Swanson, Nolan, and Pelham Rating Scale (SNAP-IV) [15] were filled by the parents, confirming the diagnosis and a significant level of symptoms in terms of inattention as well as of hyperactivity and impulsivity (CPRS: Cognitive problems due to attention deficit raw score 17 (T score: 75), hyperactivity raw score 19 (T score: 74), ADHD symptoms raw score 24 (T score 77); SNAP-IV: attention deficit 2.12 (cut off 1.78), hyperactivity 2.33 (cut off 1.44), no significant oppositional-defiant functioning). The final diagnosis was, therefore ADHD, combined presentation, moderate-to-severe.

Parents, who had previously searched the Internet for information, requested the possibility to start a pharmacological treatment to help their child. Due to existing regulations for the prescription of methylphenidate in Italy, however, we had to deny their request due to the child’s age (as he was less than six years old). We proposed as an alternative, at least pro-tempore, the use of PUFAs; the parents accepted this, although not happily (as they had read of the effects of methylphenidate and had great expectations from its use in G.).

G. was administered eicosapentaenoic acid (EPA) 558 mg + docosahexaenoic acid (DHA) 174 mg + gamma-linolenic acid (GLA) 60 mg per day in the form of chewable capsules taken twice per day. The parents were informed that it was necessary to allow a sufficient amount of time before assessing the efficacy of this approach. An appointment was booked after two months of treatment. We provided counselling both to the parents and the teachers, and scheduled periodic monitoring phone contacts.

The first month was described as difficult, possibly because parents and teachers were more informed and tended, therefore, to observe and report more behaviors typical of ADHD. Then, in the parent’s words, “he started to change” towards a better functioning: He was able to pay attention for a longer time both at kindergarten and at home, he was easier to stop when starting to be too active, and had less tendency to intrude on other’s conversations. His drawing skills started to improve, possibly as a consequence of a much longer exercise and attention paid to the task. The general pediatrician confirmed the parent’s reporting of a significant reduction of the previously frequent minor injuries following motor hyperactivity.

All questionnaires and interviews showed a reduction of both symptoms and impairment (CPRS: cognitive problems due to attention deficit raw score 14 (T score: 68), hyperactivity raw score 16 (T score: 68), ADHD symptoms raw score 21 (T score 72); SNAP-IV: attention deficit 1.89.12 (cut off 1.78), hyperactivity 2 (cut off 1.44)), so that the severity of expression was changed to moderate. We suggested to maintain the treatment as in use, a fact that the parents were at this time happy to accept.

Treatment was continued for six months, without any significant side effect; G. only reported to prefer not to chew the capsule because “they don’t taste good”. The improvement was lasting and substantially stable. No longer was follow up possible because the family moved quite far from our medical center.

### 2.2. Case 2—Dysregulated but Not Specific

M. was seen at the age of eight years and three months. His parents reported significant concerns expressed by his teacher: He was not obtaining expected results, he was messy and his handwriting was hard to read, despite his efforts and some specific training provided by the school and the parents. Moreover, according to the parents he was often a bit slow in following orders, despite not refusing to obey. He had some “tantrums”, appearing suddenly and without connection with significant events, and sometimes his mood was unstable.

He was a second son and had no significant family history of any neuropsychiatric disorder. Pregnancy was described as fully normal, while birth had been characterized by a delay of the expulsive phase, without detectable signs of perinatal brain problems. He had reached developmental milestones in typical times, although parents described him as always clumsy and somewhat “unstable” in his behavior.

The clinical examination was in line with the description provided. Cognitive functioning was normal, but writing skills and global praxic abilities were slightly below average when formally tested; he was able to stay focused for an adequate time, but his ability to shift attention to a target proposed by the examiner was below average. During the testing, he had two moments of loss of emotional control, apparently caused by minor problems (one time he misread a word, the other time apparently his drawing was not up to his expectations). All his neuropsychological parameters related to academic skills (i.e., reading fluency and correctness, writing fluency and correctness, ability to manipulate numbers and to calculate) were at the lower limit of the normal range (indicating a widespread frailty of these aspects without a significant deficit in any of them).

A diagnosis of “mixed specific developmental disorder” was proposed, with an added indication of a frailty in emotion regulation. The parents, preoccupied with the possibility of academic failure, accepted the proposal of a psychomotor treatment aiming at improving praxic skill, but posed the question of the possibility to support this with a pharmacological approach to make M. “more stable”.

Parents were offered a structured counselling to help them cope with the peculiarities of their child, but given their explicit request, M. was also started eicosapentaenoic acid (EPA) 837 mg + docosahexaenoic acid (DHA) 261 mg + gamma-linolenic acid (GLA) 90 mg per day in the form of capsules taken twice per day.

The child was re-evaluated after four months of treatment. Reports by the parents, the teacher and the general pediatrician were highly consistent in describing a significant improvement. The frequency of tantrums decreased from the third month of treatment. Academic and praxic skills began to increase from the second month of treatment (it was not possible to differentiate the effect of the psychomotor treatment from that of PUFA supplementation). The child remained able to focus his attention, but became “easier to guide” towards adult-established goals.

Treatment with PUFAs was continued for six months, then a four-months break was given, followed by a second period of treatment (this time lasting two months). Benefits obtained were stable, both during the suspension and after the second period of treatment. The child was able to improve his proficiency at school although with some aspects of “slowness” which were easily managed with didactic strategies. No side effects were reported. As a precaution, a blood sample was collected and all exams executed were unremarkable.

### 2.3. Case 3—Everything but Drugs, Please!

At the age of nine years and two months F. was sent for a consultation by the general pediatrician, who also wrote a short note describing the typical symptoms of ADHD he had observed and that had already been confirmed by both parents and teachers. F. was described as a restless child, unable to stay still or to keep sitting for more than a minute or so. He was also unable to pay sufficient attention to any activity, therefore, not completing his schoolwork nor any task proposed by his parents and adequate for his age. He was unable to make friends, because he easily got distracted during games and was therefore rejected as a “sandbag”.

Term born after a fully normal pregnancy, F. had been always seen as a child with poor attentive skills and high level of motor activity. As far as the parents could tell, he had always been seen as needing significant attention from the adults to be kept focused on the ongoing activities and to prevent him from moving around without an apparent reason but to keep moving. His father declared that he expected F. to improve spontaneously. The mother explained that almost all males in the paternal family were roughly similar to F., although apparently with less severe behaviors.

K-SADS-PL (DSM-5 version) [13] was administered and a diagnosis of Attention-Deficit/Hyperactivity Disorder was confirmed. Conners’ Parent Rating Scales [14] and Swanson, Nolan, and Pelham Rating Scale [15], filled by the parents, evidenced a significant level of symptoms in terms of hyperactivity and inattention (CPRS: cognitive problems due to attention deficit raw score 22 (T score: 73), hyperactivity raw score 15 (T score: 73), ADHD symptoms raw score 25 (T score 78); SNAP-IV: attention deficit 2.33 (cut off 1.78), hyperactivity 2.56 (cut off 1.44), no significant oppositional-defiant functioning). Global cognitive functioning was in the normal range as well as learning skills. The final diagnosis was, therefore, ADHD, combined presentation, moderate-to-severe.

Given the impact that ADHD symptoms were having on F.’s life, methylphenidate was offered, in the context of a more global intervention including parent and teacher training. Indication and possible side effects of this approach were thoroughly discussed. However, possibly after looking for additional information on the Internet, parents refused consent to the administration of methylphenidate, expressing their preoccupation with the biochemical similarities between methylphenidate and cocaine.

At the same time, however, parents expressed their preoccupation with the time needed for psychoeducational interventions to be effective and asked for alternative solutions. Since they were ready to accept it, F. was started eicosapentaenoic acid (EPA) 837 mg + docosahexaenoic acid (DHA) 261 mg + gamma-linolenic acid (GLA) 90 mg per day in the form of capsules taken twice per day.

Teachers were the first to evidence some improvements after 2.5 months: F. was described as more able to participate to everyday school activities, with a small but clear improvement in his academic results. One month later, he received his first invitation for a sleepover with his newly acquired “best friend” and after five months of treatment he was accepted in a local soccer team, where he was described by his coach as “a nice lad, even if not always focused during the matches, when his fellows have to help him sometimes”.

Treatment was continued with the same dose for one year. Parents recognized that the use of PUFAs had been useful and without side effect, but decided to change their mind as to the use of methylphenidate because they wanted to try it before their child becoming an adolescent. The administration of the drug under medical control (so called “dose test”) provided clear evidence of efficacy and methylphenidate was then started following standard protocols in keeping with the Italian laws (i.e., a therapeutic plan was defined by our Center and regularly monitored with clinical, ECG, blood examinations as prescribed by the Italian laws concerning the use of methylphenidate and of other drugs to treat ADHD). Following parental request, PUFAs were suspended.

### 2.4. Case 4—Methylphenidate Is Not Enough

F. (the patient whose story was previously reported as “case 3”) had a significant improvement using methylphenidate. He was started using immediate-release methylphenidate, first once per day and then twice per day. After a couple of months, he was given delayed-release methylphenidate to optimize the efficacy and to eliminate the need of a second dose after lunch (which required the parents to go to school to have F. take his pills, as the school had no nurse or similar facilities).

As F. grew, the dose of methylphenidate was adapted to his weight and kept around 0.5 mg/kg/dose (or equivalent for delayed-release methylphenidate). When F. reached 12 years and eight months, parents started to report some unusual movements of the shoulders. During the neurological examination, this was understood as a motor tic and other tics were noted, both motor (eye blinking, movements of the wrists) and occasionally vocal (one episode of repetitive throat clearing, without evidence of any relevant medical condition). Since this was a possible side effect of methylphenidate, and considering parental concerns (both in the past and at present) towards this drug, the dosage was reduced to the equivalent of 0.4 mg/kg/dose. This led to an almost immediate disappearance of tics, but also to a reduction of the positive effects of methylphenidate: F. was described as less able to pay attention, with an apparent shorter efficacy of the delayed-release formulation he was taking.

After discussion with the parents, it was decided to re-introduce PUFAs in an attempt to strengthen the effect of methylphenidate without increasing the pro-kilo dose of the drug. F. was started eicosapentaenoic acid (EPA) 558 mg + docosahexaenoic acid (DHA) 174 mg + gamma-linolenic acid (GLA) 60 mg per day in the form of chewable capsules taken twice per day. After about two months of this regimen, all adults around F. (parents, teachers, soccer coach) agreed that his functioning was almost identical to that seen when he was taking a higher dose of methylphenidate.

Both methylphenidate and PUFAs were continued for roughly one year, without further side effects. Since F. had improved, a suspension was attempted and was successful, since F. was able to continue his life no longer needing pharmacological or nutraceutical support.

## 3. Conclusions

The cases depicted are examples of clinical situations, frequently occurring in real-life pediatric and neuropsychiatric practice, where the use of PUFAs contributed to the results obtained as a significant part of a multi-modal therapeutic strategy. They are not meant to demonstrate the utility of PUFAs in these situations, which has been studied in far more detail [16]. They are intended to offer real-life examples about how to use them in lack of published guidelines.

To summarize, the clinical situations were as follows:

(1) when a treatment could be useful, but the standard drug (i.e., methylphenidate) cannot be prescribed due to existing regulations;
(2) when the clinical situation is atypical, but dominated by dysregulation (both of mood and behavior);
(3) when parents refuse consent to administration of methylphenidate, despite medical advice suggesting to use it; and
(4) when methylphenidate is effective but not fully, and dose cannot be safely increased.

Given that PUFA administration is a highly safe intervention, it is important for the general pediatrician, to be aware of this option, of its possible use, of the differences existing between different products in order to best serve the interest of the patient and of his/her family, considering the increasing use of nutraceuticals in psychiatric e neuropsychiatric disorders [17]. It is also important to consider PUFAs as part of a more comprehensive approach towards neurodevelopmental difficulties, as evidenced by the clinical vignettes described. We used a formulation providing a specific ratio (9 EPA:3 DHA:1 GLA), in line with existing studies evidencing its peculiar efficacy [18], its effect on neuronal viability [19] and its ability to improve fatty acid plasma profile [20]. Given their “real-life” nature, these case reports could be a useful source of inspiration despite the limitation connected with the nature of the case reports and with the lack of published guidelines.

## Data Availability

Raw data are available from the author upon reasonable request.
